# Discrimination and common mental disorder among migrant and ethnic groups: findings from a South East London Community sample

**DOI:** 10.1007/s00127-016-1191-x

**Published:** 2016-02-13

**Authors:** S. L. Hatch, B. Gazard, D. R. Williams, S. Frissa, L. Goodwin, SELCoH Study Team, M. Hotopf

**Affiliations:** Department of Psychological Medicine, Institute of Psychiatry, Psychology & Neuroscience, King’s College London, 10 Cutcombe Road, London, SE5 9RJ UK; Department of Social and Behavioral Sciences, Harvard School of Public Health, 677 Huntington Ave, Boston, MA 02115 USA

**Keywords:** UK, Mental health, Discrimination, Migration, Ethnicity

## Abstract

**Purpose:**

Few studies have examined discrimination and mental health in the UK, particularly by migrant status and in urban contexts with greater demographic diversity. This study aims to (1) describe the prevalence of discrimination experiences across multiple life domains; (2) to describe associations between discrimination experiences and common mental disorder (CMD); (3) to determine whether or not the relationship between discrimination and CMD varies by migrant status and ethnicity.

**Methods:**

Data on major, anticipated and everyday discrimination and CMD symptoms were collected from an ethnically diverse prospective sample of 1052 participants followed up from 2008 to 2013 in the South East London Community Health study, a population-based household survey.

**Results:**

With few exceptions, discrimination was most prevalent among those in the Black Caribbean group. However, those in the White Other ethnic group had similar or greater reporting major and anticipated discrimination to Black or mixed ethnic minority groups. The effects of discrimination on CMD were most pronounced for individuals who had recently migrated to the UK, an ethnically heterogeneous group, and for Black and Mixed ethnic minority groups in partially adjusted models. Prior CMD accounted for differences between the Mixed and White British ethnic groups, but the strength of the association for the most recent migrant group and the Black ethnic groups remained two or more times greater than the reference groups.

**Conclusions:**

The strength of the relationship suggests a need for more consideration of migration status along with ethnicity in examining the impact of discrimination on mental disorder in community and clinical samples.

**Electronic supplementary material:**

The online version of this article (doi:10.1007/s00127-016-1191-x) contains supplementary material, which is available to authorized users.

## Introduction

It is increasingly recognized that discrimination is an established contributor to poor mental health and may be a key determinant of health disparities between different socio-economic and demographic groups [1, 2]. Results from recent meta-analytic reviews of cross sectional and longitudinal studies utilising survey and experimental methods demonstrate stronger effects of discrimination on mental than physical health [1, 2]. Discrimination is a type of stressful life experience that, like other stressors, can be characterized as acute life events (major experiences of unfair treatment) and as chronic daily hassles (everyday discrimination or “micro-aggressions”) [2–5]. There is strong objective evidence of widespread differences in exposure to discrimination according to socio-economic status, race, gender and migrant status. Experimental studies demonstrate that discrimination exists; this evidence derives from both isolated (e.g., facing actual negative treatment) and pervasive (e.g., submission of comparable job applications that vary by ethnicity) manipulations [2, 6, 7]. Research on discrimination usually relies on recall of discrimination as a subjective experience [8]. However, regardless of its subjective nature, discrimination has been widely demonstrated to elicit psychological and physiological stress responses that influence health [2, 9].

Past discrimination experiences and/or the perceived threat of discrimination can also provoke behavioural and emotional responses characterised by high levels of mistrust, fear, chronic worry and rumination [10–13]. For many, this can result in anticipation of discrimination, which involves people avoiding opportunities, situations and places where they may be more vulnerable [11, 12, 14]. This process of vigilance and expectations management is likely to be based on one’s own previous experience or the experiences of family members and others within their social network [11, 14]. In addition to evidence from a small number of clinical studies, evidence from experimental studies has demonstrated that anticipated discrimination provokes potentially harmful physiological responses (e.g., increased blood pressure, heart rate and stress hormones) [10, 11, 15]. Moreover, findings from a US community population study reveal that active vigilance in relation to discrimination is a risk factor for common mental disorder (CMD; symptoms of depression and anxiety) and may also conceal underlying mental health inequalities [12]. However, ethnic identity (i.e., identification with one’s ethnic group) has been posited as having a protective effect that potentially decreases the impact of discrimination on poor mental health [1].

There is little information from UK populations about discrimination experiences and few UK studies represent multicultural, urban contexts where people are more likely to encounter demographic diversity. Many UK studies have focused on a single life domain despite there being multiple key life domains (e.g., education, health, work, housing etc.) in which discrimination takes place [16, 17]. Most UK studies of adult populations have focused on understanding discrimination or harassment based only on race, religious or ethnic background [18, 19]. However, there are many socially disadvantaged statuses (e.g., migrant status) that impact on mental health [20, 21]. Moreover, previous UK studies have not accounted for prior poor mental health in examining this association.

As with other types of stressors, discrimination experiences are shaped by structural factors (e.g., social ideologies, institutions and policies), individual social position, demographic characteristics and geographic context [22, 23]. As a result, the demography of discrimination fluctuates as the basis of discrimination shifts or becomes more salient. For example, in the context of immigration policies becoming a contentious political and social issue in many countries, migrant groups potentially face increased exposure to stigma and discrimination [24]. Migration status, used here in the broadest sense to refer to residing outside of the country of birth, is an important dimension of variation within ethnic groups but has received little attention in UK discrimination research [25, 26]. Given that 75 % of adults recently surveyed in the UK view immigration as a problem, migrants (representing 12 % of the UK and 40 % of the London population) are a group at high risk of exposure to discrimination [27, 28]. Prior research provides an unclear pattern of discrimination by migration status; some studies find that migrants report lower levels of discrimination than the native population, while others suggest that discrimination does not differ by migration status [29–31]. Moreover, migration status is associated with psychiatric disorders and reduced functioning due to poor mental health [25, 32]. The widely observed decline in migrant mental and physical health (from being initially better than the native population) is particularly pronounced among those who have resided in their new country of residence for longer [25, 26, 33]. While prior research has looked at the prevalence of discrimination by migration status, previous studies have not examined whether or not the effects of discrimination on mental health may differ by migration status.

The purpose of our study was to determine the extent to which migrants experience discrimination in a multi-ethnic urban sample. We aim to describe the patterns of discrimination by ethnicity and migration status and assess the relationship between discrimination and CMD. Given the evidence from previous studies, we hypothesise that discrimination will have a greater impact for migrant groups that have resided in the UK for longer. In contrast to previous UK studies, we examine discrimination across multiple life domains, control for prior CMD and consider a level of CMD symptoms that indicates a greater likelihood to require treatment.

## Methods

### Sample and procedure

The South East London Community Health (SELCoH) study is an UK psychiatric and physical morbidity survey of 1698 adults, aged 16 years and over residing in 1075 randomly selected households in the boroughs of Southwark and Lambeth [34]. Following SELCoH 1 (2008–2010), SELCoH 2 targeted 1596 (94 %) of participants from who agreed to be re-contacted from 2011 to 2013. Of those participants, 157 were ineligible due to death/poor health/relocation; and interviews were conducted with 1052 participants (73 % response rate) using a computer assisted interview schedule; 1022 were face to face interviews and 30 (2.9 %) were computer assisted telephone interviews for those temporarily located outside of London during data collection.

Similar to the UK National Psychiatric Morbidity Survey methods, households were identified through stratified random sampling addresses from the UK Small User Postcode Address File, which has near complete coverage of private households [35]. Introductory letters describing the study were sent and followed by up to four visits at different times of the day. Trained interviewers consented and interviewed as many eligible household members (adults aged 16 years and over) as possible; interpreters were available where necessary. Response rates for SELCoH 1 were 51.9 % household participation and 71.9 % participation within households. The overall sample was similar to the 2011 UK Census demographic and socioeconomic indicators for the catchment area (see Supplementary Table 1). Full details of the methods and sample description were previously reported [34].

Ethical approval for SELCoH 1 was received from the King’s College London Research Ethics Committee for non-clinical research populations (reference CREC/07/08-152) and for SELCoH 2 was received from the King’s College London Psychiatry, Nursing and Midwifery Research Ethics Committee (PNM/10/11-106).

### Measures

#### Common mental disorders

For SELCoH 1 and 2, common mental disorder (CMD) was assessed by the Revised Clinical Interview Schedule (CIS-R), a structured interview that asks about 14 symptom domains: fatigue, sleep problems, irritability, worry, depression, depressive ideas, anxiety, obsessions, subjective memory and concentration, somatic symptoms, compulsions, phobias, physical health worries and panic [36]. A total CIS-R score of 12 or more is used to indicate the overall presence of CMD, with a total score of 18 or more that denotes a symptom level likely to require treatment. The CIS-R also provides ICD-10 diagnoses for six mental disorders through a standard algorithm; depressive episode, generalised anxiety disorder mixed anxiety and depressive disorder were most prevalent in the SELCoH sample.

Major experiences of discrimination was a structured set of items that asked participants if they have ever (yes/no) been unfairly fired; not hired for a job; denied a promotion; stopped, searched, questioned, physically threatened or abused by the police; treated unfairly in the court system; treated unfairly when getting medical care (in mental/physical health care); treated unfairly when using public transportation; unfairly discouraged by a teacher or advisor from continuing education; unfairly prevented from moving into a neighbourhood because the landlord or a leasing agent refused to sell or rent a house or apartment; unfairly treated by neighbours who made life difficult for yourself or family; unfairly denied a bank loan or received a less preferable mortgage rate; received service from someone (e.g., plumber or car mechanic) that was worse than others would receive [16]. This measure was similar to Williams et al. [31], with the exception of added court system and public transportation domains.

Every day discrimination was measured with ten items capturing how often on a day-to-day basis participants experienced being treated with less courtesy than others; being treated with less respect than others; receiving poorer service than others in stores/restaurants; people acting as if they were not smart; people acting as if they are afraid of them; people acting as if they think they are dishonest; people acting as if they are inferior; being called names or insulted; being threatened or harassed and being followed around in stores [16]. The last item was added to the original measure and the internal consistency of the scale remained high (*α* = 0.86). This addition was confirmed with factor analysis with all items loading highly onto a single factor (≥0.60). We dichotomised the responses into often/sometimes versus almost never/never and summed to compare scores at and above the median versus below the median (range 0–10; median 6.0; weighted mean 6.5).

Anticipated discrimination items were taken from the Discrimination and Stigma scale (DISC) [14] and were modified to capture to what extent participants have stopped themselves from applying for work or for training/education; contacting health services; and going into certain areas/neighbourhoods. The items were considered separately and not summed. We dichotomised the responses into a little/somewhat/a lot versus not at all after detecting some skewness with the distribution.

Ethnicity refers to a self-reported UK Census category; the Mixed ethnic group includes combinations of Black, Asian and White groups; the Non-White Other group includes Indian, Pakistani, Chinese, Latin American and other Black and Asian groups; and the White Other group primarily includes participants from other European countries and North Africa. Migration status refers to the number of years residing in the UK, accounting for information provided at SELCoH 1 and timing up to SELCoH 2. Ethnic identity was captured using the Multigroup Ethnic Identity Measure (MEIM), 20 items that capture identification with and belonging to one’s own ethnic group rated on Likert-type responses ranging from strongly disagree to strongly agree [37]. Items are summed and the mean is obtained; scores range from 1 to 4 (*α* = 0.83). Other potential confounders reported at SELCOH 2 included age; gender; sexual identification (non-heterosexual and heterosexual); relationship status; highest education level; employment status and English as a first language reported at SELCoH 1 (detailed in Table 1).

**Table 1:** Sample characteristics and prevalence of common mental disorder by ethnicity and migration status (*N* = 1052)

	Total sample, *n* (%)	Migration status Number of years in the UK					Ethnic identity^a^	Common mental disorder	
		≤10 years, *n* (%)	11–20 years, *n* (%)	≥21 years, *n* (%)	UK born, *n* (%)	*p* value	Mean score (SD)	CIS-R score ≥12, *n* (%)	*p* value
Ethnicity									
White British	536 (49.7)	2 (0.4)	6 (1.3)	16 (2.9)	500 (95.4)	0.001	2.6 (0.51)	109 (20.7)	0.26
Black African	135 (13.6)	38 (29.2)	37 (27.4)	30 (17.9)	30 (25.5)		3.3 (0.47)	25 (18.4)	
Black Caribbean	85 (8.3)	6 (7.4)	10 (11.8)	17 (15.4)	52 (65.3)		3.3 (0.39)	19 (21.7)	
Mixed^b^	50 (5.4)	6 (11.0)	7 (15.2)	4 (6.1)	33 (67.6)		2.7 (0.61)	10 (18.6)	
Non-White Other^c^	98 (9.5)	31 (34.4)	19 (17.6)	26 (22.8)	21 (25.2)		3.0 (0.52)	27 (27.8)	
White Other^d^	147 (13.6)	43 (30.1)	31 (22.2)	40 (23.5)	31 (24.2)		2.8 (0.57)	41 (28.2)	
Number of years in UK									
UK born	668 (65.4)							142 (21.5)	0.23
≥21 years	133 (10.8)							37 (28.6)	
11–20 years	110 (10.9)							27 (25.4)	
≤10 years in the UK	126 (12.8)							23 (18.0)	
English as first language									
No	214 (19.8)							48 (21.9)	0.97
Yes	838 (80.2)							183 (22.1)	
Age (in years)									
17–35	270 (33.2)								
36–54	509 (46.3)								
55+	273 (20.4)								
Gender									
Female	615 (52.5)								
Male	437 (47.5)								
Sexual identification									
Non-heterosexual	67 (6.8)								
Heterosexual	985 (93.1)								
Relationship status									
Not in a relationship	485 (48.9)								
Married or cohabitating	567 (51.1)								
Educational level									
No qualifications	92 (7.7)								
GCSE or equivalent	168 (15.6)								
A level	262 (26.5)								
Degree or above	530 (50.2)								
Employment status									
Not in employment	413 (39.9)								
In employment	638 (60.1)								
Common mental disorder									
CIS-R score 12–17	102 (9.8)								
CIS-R score ≥18	129 (12.3)								

Weighted percentages are presented to account for survey design; frequencies are unweighted and may not add up due to missing values

^a^Ethnic identity score range 1–4

^b^Mixed ethnic group includes any combination of Black, Asian and White ethnic groups

^c^Non-White other ethnic group includes Indian, Pakistani, Chinese, Latin American and other Black and Asian groups

^d^White Other ethnic group primarily includes participants from North Africa and other European countries

#### Analytic strategy

Analyses were conducted in STATA 11 and we used survey commands to account for household clustering and to generate robust standard errors [38]. We used weights for within household non-response and sample attrition. We report the unweighted frequencies and weighted percentages and 95 % confidence intervals (CIs). Odds ratios (OR) with 95 % CIs are presented for logistic regression models and relative risk ratios (RRR) with 95 % CIs are presented for multinomial logistic regression models. For domain specific and cumulative discrimination models in Tables 3 and 4, CMD symptoms across three categories: 0–11 as the reference, 12–17 indicating moderate CMD levels and 18 or more representing a symptom level likely to require treatment. Post-estimation comparisons across ethnicity and migrant status groups in Table 5 are presented for logistic regression models testing associations between lifetime discrimination and CMD, as indicated by CIS-R scores ≥12 due to small cell sizes. Supplementary analyses are presented for the number of major discrimination domains and three primary ICD-10 diagnoses generated by the CIS-R (Supplementary Table 2). Partially adjusted models adjusted for age (continuous), gender, sexual identification, ethnicity, migration status, relationship status, education, employment status, and ethnic identity. The final models include further adjustment for CMD at SELCoH 1 to account for prior mental health.

## Results

Approximately 35 % of the SELCoH 2 sample was born outside the UK and approximately 50 % of the sample identified as being members of ethnic minority groups (Table 1). The mean age was 43.6 years (SD 16.6) years and approximately 40 % were not in employment; the majority of the sample were women and had obtained educational qualifications. While the distribution of migrant status indicated by years in the UK was evenly distributed across Black African, Non-White Other or White Other groups, 65.3 % of the Black Caribbean and 67.6 % of the Mixed groups were born in the UK. The prevalence of CMD symptoms was 22.1 % for the total sample for SELCoH 2, with 12.3 % having a CIS-R score ≥18. Approximately 12.5 % met the criteria for CMD at both time points (not shown). Depressive episodes (10.7 %), generalised anxiety disorder (6.9 %) and mixed anxiety and depression (6.1 %) were the most common primary ICD-10 diagnoses (not shown). There was no difference in CIS-R scores by ethnicity, number of years in the UK or English as first language.

### Discrimination by ethnicity and migration status

In the total sample, major discrimination from the police, potential employers (not hired) and educators were the most commonly reported (Table 2). Across many domains, Mixed, Black Caribbean and White Other groups were the most likely to report major discrimination. Notably, those in the White Other group, of which 59.2 % identified as White European, had similar or greater proportions of reported major discrimination across most of the domains than other ethnic minority groups. The Black Caribbean group was more likely to report police-related discrimination than other ethnic groups, with the exception of the Mixed group. A greater proportion of those in the Black Caribbean and Mixed groups reported education-related discrimination than the White and Non-White Other groups. Discrimination related to not being hired for a job was most commonly reported by Black Caribbean and Black African groups; anticipating discrimination in education and employment was two times greater in these groups in comparison to other ethnic groups. In terms of migration status, discrimination related to the employment domain (i.e., not being hired; being fired; denied promotion) was most commonly reported among migrant groups.

**Table 2:** Prevalence of lifetime major discrimination events by socio-demographic and socioeconomic indicators (*N* = 1052)

Major discrimination	In education (%)	Fired (%)	Not hired (%)	Denied promotion (%)	In police treatment (%)	In court system (%)	In medical care (%)	In public transport (%)	In housing (%)	By neighbours (%)	In bank services (%)	In general services (%)	At least one domain (%)
Total sample	11.9	10.3	12.9	9.4	16.7	5.3	5.7	8.9	1.7	8.6	4.2	7.5	49.3
Ethnicity													
White British	10.1	8.9	8.7	5.8	12.9	5.2	4.5	7.5	0.8^d^	7.4	3.2	7.2	44.1
Black African	13.9	9.1	19.2	12.4	18.9	4.3	2.8^d^	10.5	4.6	10.0	5.3	6.5	59.2
Black Caribbean	21.4	14.1	21.3	17.4	33.9	9.7	5.9	9.1	0.8^d^	8.6	6.8	6.3	64.9
Mixed^a^	25.9	5.1^d^	13.9	9.9	28.6	6.4^d^	10.3	16.8	5.9^d^	19.8	2.4^d^	8.6^d^	57.0
Non-White Other^b^	4.6	9.2	14.9	8.1	13.3	2.1^d^	8.3	4.6^d^	0.0^d^	4.7^d^	3.1^d^	6.6	37.9
White Other^c^	10.1	16.4	14.2	15.6	15.3	5.1	8.3	11.5	2.4^d^	10.3	6.6	9.3	53.6
Number of years in UK													
UK born	13.7	10.0	10.3	7.6	18.1	5.3	5.2	9.1	1.1	8.9	3.4	7.5	48.5
≥21 years	8.6	10.7	14.1	17.6	11.9	6.7	4.5	5.0	2.7^d^	8.6	7.8	10.9	51.6
11–20 years	10.1	7.6	19.9	10.7	21.9	5.9	8.2	9.6	3.9^d^	9.7	5.8	4.1	56.5
≤10 years	7.8	13.7	20.4	11.4	9.1	2.8^d^	6.3	11.6	2.5^d^	5.9	4.1	7.2	45.7

Anticipated discrimination		Avoid applying for education or work			Avoid health service contact		Avoid certain neighbourhoods		At least one domain					
Total sample		14.2			3.9		18.9			28.9				
Ethnicity														
White British		10.0			3.2		16.3			24.2				
Black African		25.0			3.2		20.4			36.7				
Black Caribbean		27.2			3.1^d^		26.9			41.1				
Mixed^a^		9.8			4.0^d^		22.7			28.4				
Non-White Other^b^		14.6			7.8		22.7			34.2				
White Other^c^		12.2			3.8		18.2			27.5				
Number of years in UK														
UK born		12.3			3.7		19.0			28.1				
≥21 years		16.1			3.9		16.2			27.8				
11–20 years		16.9			5.3		16.6			27.9				
≤10 years		20.6			3.4		23.6			35.9				

^a^Mixed ethnic group includes any combination of Black, Asian and White ethnic groups

^b^Non-White Other ethnic group includes Indian, Pakistani, Chinese, Latin American and other Black and Asian groups

^c^White Other ethnic group primarily includes participants from North Africa and other European countries

^d^Cell size < 5

Avoiding certain neighbourhoods was the most commonly reported anticipated discrimination domain for the total sample; there was no difference by ethnicity or migration. In contrast, avoiding health care was more likely to be reported by the Non-White Other group. Among migrant groups, anticipated discrimination in education/work was more common among UK residents of 10 years or less.

The everyday discrimination weighted mean scores were significantly higher among Black Caribbean (9.1; *p* value = 0.001) and Black African (8.2; *p* value = 0.03) groups than the White British (6.7) group; no difference was found for White Other (6.4) and Non-White Other (6.4) groups (not shown). No difference was found for everyday discrimination scores by length of UK residence.

### Associations between discrimination and CMD

With the exception of discrimination related to housing, there was an approximately two-fold or greater increase in the odds of meeting the criteria for CMD at either the moderate or more severe level across domain types in unadjusted models (Table 3). In the final model that included adjustments for CMD at SELCoH 1, these associations were fully attenuated only for discrimination related to being fired and treatment by the police. For any major and anticipated discrimination, there was no association in the adjusted model at the moderate level of CMD (CIS-R scores of 12–17), but this association remained for those with CIS-R scores of 18 or more with minimal attenuation in the adjusted model. In contrast, the relationship between everyday discrimination and both levels of CMD persisted in the adjusted model with little or no attenuation.

**Table 3:** Odds of common mental disorder at SELCoH 2 by type of discrimination events

	Common mental disorder at SELCoH 2^a^					
	Model 1			Model 2		
	Ref.^a^	CIS-R score 12–17	CIS-R score 18+	Ref.^a^	CIS-R score 12–17	CIS-R score 18+
		Unadjusted RRR (95 % CI), *p* value	Unadjusted RRR (95 % CI), *p* value		Adjusted RRR^b^ (95 % CI), *p* value	Adjusted RRR^b^ (95 % CI), *p* value
Major discrimination event						
Any major discrimination event	1.0	1.5 (1.0–2.3), 0.05	2.7 (1.8–4.0), < 0.001	1.0	1.4 (0.9–2.2), 0.11	2.4 (1.5–3.8), <0.001
Fired	1.0	1.8 (0.9–3.3), 0.06	2.0 (1.2–3.4), 0.01	1.0	1.3 (0.7–2.6), 0.38	1.3 (0.6–2.6), 0.50
Not hired	1.0	2.4 (1.4–4.2), 0.001	1.4 (0.8–2.4), 0.20	1.0	2.6 (1.4–4.8), 0.002	1.1 (0.5–2.2), 0.76
Denied promotion	1.0	2.3 (1.2–4.1), 0.01	2.1 (1.1–3.7), 0.01	1.0	2.6 (1.4–4.8), 0.004	1.8 (0.8–3.9), 0.12
In police treatment	1.0	0.9 (0.5–1.6), 0.66	1.7 (1.0–2.8), 0.03	1.0	0.9 (0.4–1.8), 0.80	1.8 (0.9–3.5), 0.08
In court system	1.0	1.3 (0.5–3.4), 0.53	4.2 (2.2–7.9), <0.001	1.0	0.7 (0.2–2.2), 0.51	2.5 (1.1–5.7), 0.02
In education	1.0	1.7 (0.9–3.2), 0.07	2.8 (1.7–4.7), <0.001	1.0	1.2 (0.6–2.5), 0.53	2.2 (1.1–4.3), 0.02
In housing	1.0	2.0 (0.5–7.6), 0.30	2.6 (0.8–8.5), 0.11	1.0	1.2 (0.3–5.8), 0.78	0.5 (0.9–3.2), 0.51
By neighbours	1.0	1.2 (0.6–2.7), 0.58	3.3 (1.9–5.6), <0.001	1.0	0.9 (0.4–2.2), 0.91	2.2 (1.1–4.3), 0.02
In bank services	1.0	2.0 (0.8–5.0), 0.11	3.7 (1.8–7.6), <0.001	1.0	2.3 (1.0–5.3), 0.04	4.1 (1.7–9.5), 0.001
In general services	1.0	2.0 (1.0–3.9), 0.04	2.4 (1.3–4.3), 0.003	1.0	2.1 (1.0–4.4), 0.04	2.7 (1.3–5.6), 0.01
In health services^c^	1.0	2.5 (1.1–5.5), 0.02	3.7 (2.1–6.8), <0.001	1.0	1.9 (0.7–4.9), 0.18	2.9 (1.4–5.8), 0.004
In public transport	1.0	2.7 (1.4–5.1), 0.003	3.6 (2.1–6.1), <0.001	1.0	2.0 (0.9–4.2), 0.06	3.0 (1.5–5.8), 0.001
Anticipated discrimination						
Any anticipated discrimination event	1.0	1.7 (1.1–2.7), 0.01	2.9 (2.0–4.4), <0.001	1.0	1.4 (0.9–2.3), 0.17	2.9 (1.8–4.8), <0.001
Avoid applying for education or work	1.0	2.0 (1.2–3.4), 0.01	2.8 (1.8–4.6), <0.001	1.0	1.8 (0.9–3.3), 0.06	2.7 (1.6–4.8), <0.001
Avoid health service contact	1.0	4.1 (1.5–11.2), 0.01	12.3 (5.9–25.3), <0.001	1.0	2.0 (0.6–6.2), 0.23	6.3 (3.0–13.0), <0.001
Avoid neighbourhoods	1.0	1.3 (0.8–2.2), 0.36	1.8 (1.1–2.8), 0.02	1.0	1.2 (0.7–2.1), 0.57	1.9 (1.1–3.5), 0.02
Everyday discrimination						
Median score and above	1.0	1.7 (1.1–2.6), 0.02	2.5 (1.6–3.8), <0.001	1.0	1.6 (1.0–2.7), 0.04	3.2 (1.9–5.3), <0.001

^a^Reference category = CIS-R score 0–11

^b^Model adjusted for age, gender, sexual identification, ethnicity, migrant status, English as 1st language, relationship status, education, employment status, ethnic identity and common mental disorder at SELCoH 1

^c^Mental and physical health care combined due to small cell size for individual items

Cumulative exposure of major discrimination illustrates a gradient in this association (test for trend *p* < 0.001), with an approximately two-fold or more difference in the odds of CMD among those who reported experiences of major discrimination across two or more domains in the partially adjusted model (Table 4). Controlling for prior CMD reduced the odds of CMD, particularly the association between those who reported discrimination in three or more domains and CIS-R scores of 18 or more. However, the effect size for these associations remained approximately two or more times greater for those reporting major discrimination in two or more domains.

**Table 4:** Prevalence of number of major discrimination domains by ethnicity and migrant status and in association with common mental disorder at SELCoH 2

	Number of Major Discrimination Domains^a^					
	None, *n* (%)	One, *n* (%)	Two, *n* (%)	Three or more, *n* (%)		
Total sample	541 (50.7)	236 (22.7)	126 (12.3)	147 (14.2)		
Ethnicity						
White British	302 (55.9)	127 (24.0)	56 (10.6)	51 (9.4)		
Black African	56 (40.8)	38 (29.0)	17 (11.7)	23 (18.5)		
Black Caribbean	31 (35.0)	21 (25.6)	14 (17.9)	19 (21.5)		
Mixed^b^	22 (42.9)	6 (11.1)	10 (22.5)	12 (23.4)		
Non-White Other^c^	63 (62.1)	15 (16.7)	9 (10.4)	11 (10.7)		
White Other^d^	67 (46.3)	29 (19.0)	20 (13.3)	30 (21.3)		
Number of years in UK						
UK born	350 (51.5)	153 (23.5)	74 (11.4)	90 (13.6)		
≥21 years	64 (48.4)	32 (24.7)	14 (10.7)	22 (16.2)		
11–20 years	50 (43.5)	26 (22.9)	20 (18.8)	14 (14.7)		
≤10 years	69 (54.3)	22 (17.9)	15 (11.6)	20 (16.2)		

Total sample	Common mental disorder at SELCoH 2^e^					
	CIS-R score 12–17	CIS-R score 18+	CIS-R score 12–17	CIS-R score 18+	CIS-R score 12–17	CIS-R score 18+
	Model 1		Model 2		Model 3	
Number of major discrimination domains	Unadjusted RRR (95 % CI), *p* value	Unadjusted RRR (95 % CI), *p* value	Adjusted RRR^f^ (95 % CI), *p* value	Adjusted RRR^f^ (95 % CI), *p* value	Adjusted RRR^g^ (95 % CI), *p* value	Adjusted RRR^g^ (95 % CI), *p* value
None	1.0	1.0	1.0	1.0		
One	1.0 (0.6–1.8), 0.99	1.7 (1.1–2.9), 0.03	1.0 (0.5–1.9), 0.98	1.9 (1.2–3.3), 0.01	1.0 (0.5–1.9), 0.98	1.8 (1.1–3.1), 0.03
Two	1.5 (0.8–2.8), 0.22	2.1 (1.2–3.9), 0.01	1.7 (0.9–3.4), 0.11	2.6 (1.4–5.0), 0.004	1.6 (0.8–3.2), 0.18	2.2 (1.1–4.5), 0.03
Three or more	2.8 (1.6–4.9), <0.001	5.5 (3.4–9.1), <0.001	2.7 (1.5–4.9), 0.001	6.3 (3.7–10.8), <0.001	2.2 (1.2–4.1), 0.01	3.9 (2.0–7.5), <0.001

^a^Domain range = 0–9

^b^Mixed ethnic group includes any combination of Black, Asian and White ethnic groups

^c^Non-White Other ethnic group includes Indian, Pakistani, Chinese, Latin American and other Black and Asian groups

^d^White Other ethnic group primarily includes participants from North Africa and other European countries

^e^Reference category = CIS-R score 0–11

^f^Model 2 is partially adjusted for age, gender, sexual identification, ethnicity, migrant status, English as 1st language, relationship status, education, employment status, ethnic identity

^g^Model 3 includes further adjustment for common mental disorder at SELCoH 1

In the fully adjusted models for the three most prevalent CIS-R primary diagnoses, the associations between any major and everyday discrimination and CMD appeared to be limited to depressive episodes in the fully adjusted models presented in Supplemental Table 2. In contrast, any anticipated discrimination was associated with depressive episodes and generalised anxiety. For major discrimination domains, discrimination in job promotions, in general services, on public transport and by neighbours were associated with depressive episodes. Anticipated discrimination in education or work was also related to this outcome. Additionally, there were domain specific associations for discrimination related to employment hiring with mixed anxiety and depression.

Table 5 presents partially and fully adjusted models for post estimation comparisons of associations between discrimination and CMD among ethnicity and migrant groups who reported any major, anticipated and everyday discrimination. In comparison to the White British group who reported discrimination, the associations with CMD were at least three times greater for the Black African, Black Caribbean and Mixed ethnic groups in the partially adjusted models. Adjusting for prior CMD reduced these associations for the Black African and Black Caribbean groups, but they remained at least two to three times greater than the White British group. Notably, the addition of prior CMD appears to result in a slight attenuation in the difference between the Black African and White British groups but not between the Black Caribbean and White British groups. In contrast, the difference between the Mixed ethnic group and the White British group was accounted for by adjusting for prior CMD. There was no difference in the effect of discrimination in relation to CMD for the Non-White Other and White Other groups compared to the reference group.

**Table 5:** Adjusted odds ratios for post-estimation comparisons of associations between discrimination and common mental disorder among ethnicity and migrant groups who reported discrimination

	Common mental disorder at SELCoH 2	
	CIS-R score ≥12	CIS-R score ≥12
	Model 1^a^	Model 2^b^
	Adjusted OR (95 % CI), *p* value	Adjusted OR (95 % CI), *p* value
Any major discrimination and ethnicity		
White British	1.0	1.0
Black African	3.6 (1.7–7.7), 0.001	2.6 (1.1–5.8), 0.02
Black Caribbean	3.9 (1.8–8.7), 0.001	3.6 (1.5–8.6), 0.01
Mixed	3.3 (1.1–8.4), 0.01	2.0 (0.8–5.2), 0.15
Non-White Other	1.3 (0.6–2.8), 0.42	0.9 (0.4–2.1), 0.89
White Other	1.4 (0.7–2.8), 0.30	1.1 (0.5–2.4), 0.84
Any anticipated discrimination and ethnicity		
White British	1.0	1.0
Black African	3.4 (1.7–7.1), 0.001	2.8 (1.3–6.0), 0.01
Black Caribbean	3.8 (1.7–8.3), 0.001	3.8 (1.6–9.3), 0.003
Mixed	3.3 (1.4–7.7), 0.01	2.2 (0.9–5.3), 0.08
Non-White Other	1.5 (0.7–3.3), 0.25	1.1 (0.5–2.6), 0.73
White Other	1.3 (0.7–2.6), 0.37	1.1 (0.5–2.4), 0.73
Any everyday discrimination and ethnicity		
White British	1.0	1.0
Black African	3.7 (1.7–7.9), 0.001	2.9 (1.3–6.4), 0.01
Black Caribbean	4.3 (1.9–9.8), < 0.001	4.3 (1.8–10.3), 0.001
Mixed	3.5 (1.4–8.9), 0.01	2.4 (0.9–6.1), 0.08
Non-White other	1.5 (0.7–3.3), 0.30	1.1 (0.5–2.6), 0.76
White Other	1.5 (0.7–2.9), 0.28	1.2 (0.5–2.7), 0.60
Any major discrimination and number of years in UK		
UK born	1.0	1.0
≥21 years	1.4 (0.7–2.8), 0.36	1.2 (0.6–2.6), 0.63
11–20 years	1.9 (0.9–4.2), 0.09	1.4 (0.6–3.2), 0.35
≤10 years	2.6 (1.1–5.9), 0.02	2.2 (0.9–5.5), 0.08
Any anticipated discrimination and number of years in UK		
UK born	1.0	1.0
≥21 years	1.4 (0.7–2.8), 0.39	1.3 (0.6–2.9), 0.46
11–20 years	1.8 (0.8–3.8), 0.13	1.5 (0.7–3.3), 0.26
≤10 years	2.7 (1.2–6.1), 0.02	2.6 (1.1–6.4), 0.03
Any everyday discrimination and number of years in UK		
UK born	1.0	1.0
≥21 years	1.7 (0.8–3.7), 0.18	1.6 (0.7–3.7), 0.23
11–20 years	1.8 (0.9–3.9), 0.11	1.6 (0.7–3.5), 0.21
≤10 years	2.7 (1.2–6.0), 0.01	2.7 (1.1–6.5), 0.02

Fully adjusted post estimation model comparisons of each category with the White British and UK born who reported discrimination events as the reference group

^a^Model 1 is adjusted for age, gender, sexual identification, ethnicity (in migration models), number of years in the UK (in ethnicity models), English as 1st language, relationship status, education, employment status and ethnic identity

^b^Model 2 includes further adjustment for common mental disorder at SELCoH 1

For migrant status groups, there appears to be a gradient that suggests that the effects of discrimination on CMD were strongest for those who had resided in the UK for 10 years or less. The association between discrimination and CMD decreased as number of years in the UK increased. In the partially adjusted models, the only difference between the groups appears to be for those who have resided in the UK for 10 years or less in comparison to the UK born. After controlling for prior CMD, differences between the most recent migrant group and the UK born group persists for the associations between any anticipated and everyday discrimination with CMD. Moreover, there is little or no reduction in the nature of the association.

## Discussion

In an urban UK community sample characterised as ethnically diverse with high rates of migration, this study illustrated that major, anticipated and everyday discrimination across a wide range of domains have negative consequences for symptoms of CMD. Further, the deleterious effects of all types of discrimination on CMD appeared to be most pronounced for individuals who have recently migrated to the UK and Black and Mixed ethnic minority groups in comparison to the UK born and White British groups. The likelihood of meeting the criteria for CMD was nearly three or more times greater for those in the most recent migrant group as well as the Black African and Black Caribbean groups in comparison to the UK born and White British groups, even after accounting for prior CMD symptoms. In contrast, prior CMD accounted for differences between the Mixed and White British ethnic groups. With few exceptions, discrimination across domains was most prevalent among those in the Black Caribbean group. However, the White Other group, an understudied and heterogeneous ethnic group, had similar or greater reporting of major and anticipated discrimination compared to Black or Mixed ethnic minority groups. In consideration of migrant status, the group who had resided in the UK 11–20 years (primarily consisting of those born in Africa and in Europe (approximately 39 and 30 %, respectively) reported more major discrimination, but it was those who have resided in the UK for 10 years or less that most commonly experienced anticipated discrimination. Despite no difference in the everyday discrimination scores by migration status, there was an association between everyday discrimination and CMD for the most recent migrant group, even after accounting for CMD at SELCoH I.

### Comparisons with previous studies

As in SELCoH 1, similar proportions of the sample met the criteria for CMD (22.9 % in SELCoH 1 and 22.1 % in SELCoH 2) [34]. Because we assessed discrimination across a wide range of domains, it is difficult to compare prevalence estimates with previous UK studies. However, using the same measures as in the SELCoH study, the prevalence of major discrimination in employment and education domains in a South London sample of service users diagnosed with a mental illness was similar to SELCoH participants who had the most severe level of CMD symptoms (CIS-R score ≥18) [39].

Despite controlling for a wider range of potential confounders, the general findings demonstrating at least a 2 to 4-fold risk for CMD among those reporting discrimination were consistent with previous UK national population studies using the same measure of CMD [18, 19]. This study added to previous findings by presenting results for CMD at a symptom level likely to require treatment and CIS-R primary diagnoses, as well as adjusting for prior CMD. Moreover, the findings indicated that the associations between any major, anticipated and everyday discrimination and CMD were related to depressive episodes. Interestingly, anticipated discrimination, an understudied area, was also associated with generalised anxiety.

As in SELCoH 1, there were no identified inequalities in CMD by ethnicity (when comparing ethnic minority groups to the White British group) or migration status [32, 34]. However, even in the absence of inequalities in mental health, discrimination has disparate effects on mental health by migrant status and ethnicity. There was a distinct effect on CMD among the most recent migrant group suggesting that the discrimination experiences of this group needs further study to distinguish from longer term residents. Previous studies have suggested that factors such as status loss and a limited social support following migration may contribute to deleterious outcomes [25]. Moreover, for many participants who have moved from a country in which they were among the ethnic majority, they may have ineffective coping strategies to confront these stressors. It is also possible that negative public attitudes towards migrants are being manifested through an increase in discriminatory behaviours, particularly in domains (e.g., employment, housing, health service) where migrant groups have been portrayed as social and economic burdens.

Previous findings indicate that increased exposure to minority status results in poorer mental health outcomes [25, 40, 41]. Despite some suggestion in this sample that the effects of discrimination for CMD potentially decreases over time, the associations for those groups residing in the UK longer are not statistically different from the UK born group. Unlike previous UK studies, we were able to account for prior CMD symptoms, and this appears to account for identified differences between the most recent migrant group and the UK born group for major discrimination but not for anticipated or everyday discrimination. This study also adds to the numerous studies that have demonstrated the impact of discrimination on mental health for Black ethnic groups, but fewer have examined this relationship for White ethnic minority groups in the UK or elsewhere [20]. While there were similarities in the prevalence of discrimination for the White Other, Black and Mixed ethnic groups, the effects of discrimination on CMD for the White Other group do not appear to be different for those in the White British group who reported discrimination. However, the ethnic composition of the most recent migrant group (32 % Black African, 31.2 % White Other and 25.6 % Non-White Other) suggests that an intersectional approach may be necessary to better understand discrimination and mental health in this population.

The relationship between anticipated discrimination and CMD, particularly for recent migrants and Black ethnic groups, were consistent with results from a US racially diverse community sample showing that vigilant anticipatory coping is associated with increased odds of depression [12]. However, there is limited evidence on the extent to which people adopt vigilance behaviours to limit exposure to discrimination. It is also not clear whether or not this anticipatory behaviour is protective or has negative social consequences by limiting opportunities (e.g., education attainment and potential employment).

### Study strengths and limitations

Among the strengths of this study is its examination of multiple types (i.e., major, anticipated and everyday) of discrimination across several life domains by ethnicity and migrant status. We are unaware of any other UK community population study that has taken such a comprehensive approach. Previous studies suggest that participants in the UK may consider this topic particularly difficult to discuss and thus, underreporting of discrimination is a possible limitation [18]. There were small cell sizes for some discrimination domains; thus, the prevalence estimates for these (as noted in the Table 2) should be considered with caution. It is also possible that the likelihood of exposure to specific domains where discrimination can occur (e.g., employment) varies across the life course [42]. While the age range of the SELCoH sample captures the transformation into adult social roles through to post-retirement, collecting lifetime exposure in adulthood may not comprehensively represent early life experiences which generate larger effects sizes for poor mental health in younger samples [2]. There are relatively few UK studies in this area, but findings from UK Millennium Cohort Study have recently shown the negative intergenerational impact of discrimination on child health and evidence from a multi-ethnic adolescent sample has shown the deleterious impact of racism on psychological distress [43, 44]. Deriving the main associations from cross sectional data may be another limitation; however, the results from a recent meta-analytic review of both cross sectional and longitudinal studies support the direction of discrimination impacting poor mental health [2]. Moreover, we were able to account for prior CMD in our models. Finally, there was greater loss to follow up among SELCoH participants who were younger, male, and unemployed. However, we retained 73 % of the sample and CMD symptom level was not a factor that predicted non-participation in SELCoH 2. Additionally, the key demographic and socioeconomic similarities remained between the SELCoH sample and the catchment area population according to the UK 2011 Census (Supplementary Table 1).

### Future directions

More studies need to consider how complex factors, such as migration status that denote substantial change or loss of status, may elucidate the impact of discrimination on mental disorder. For many, migration not only involves adversities, such as separation from family and limited socioeconomic opportunities [45], but also a shift from the ethnic majority to ethnic minority status. This gap in knowledge exists for community population samples, as well as more specific groups, such as mental health service users whose discrimination experiences across key life domains, particularly health service use, are influenced by the intersection of race, ethnicity and mental illness status [39, 46, 47]. Particular attention should be given to the extent to which anticipated discrimination is enacted in relation to accessing health services and socioeconomic opportunities. Future studies should also consider structural factors, such as anti-immigration policies, that have been shown to impact migrant mental health and health service utilisation [48, 49]. Given the strength of the relationship, further evaluation of how discrimination experiences impact mental disorder, help seeking and treatment outcomes is needed in community and clinical samples.

## Electronic supplementary material

Below is the link to the electronic supplementary material.

Supplementary material 1 (DOCX 29 kb)
