# Supplementary material for: Discrimination and common mental disorder among migrant and ethnic groups: findings from a South East London Community sample
Source: Soc Psychiatry Psychiatr Epidemiol. 2016 Feb 13;51:689–701. doi: 10.1007/s00127-016-1191-x (PMC4846681; doi:10.1007/s00127-016-1191-x)
Supplement: Supplementary file 1 — Supplementary material 1 (DOCX 29 kb) [file 127_2016_1191_MOESM1_ESM.docx]

**Supplemental Table 1. Comparisons of the SELCoH sample for wave 1 and 2 with 2011 UK census information**

|  | 2011 UK Census for the SELCoH study  catchment area^a^  n (%) | SELCoH 1 study sample  n (%) | SELCoH 2 study sample  n (%) |
| --- | --- | --- | --- |
| **Total samples** ^b^ | N=591369 | N=1698 | N=1052 |
| **Sex**  Female | 297830 (50.4%) | 959 (56.5%) | 615 (58.5%) |
| Male | 293539 (49.6%) | 739 (43.5%) | 437 (41.5%) |
| **Ethnic group**  White | 329374 (55.7%) | 1051 (63.4%) | 683 (65.0%) |
| Mixed ^c^ | 40938 (6.9%) | --- | 50 (4.8%) |
| Black-Caribbean | 46860 (7.9%) | 143 (8.7%) | 85 (8.1%) |
| Black-African | 82600 (14.0%) | 234 (13.2%) | 135 (12.8%) |
| Asian or Asian British | 35483 (6.0%) | 63 (3.5%) | 39 (3.7%) |
| Other | 56114 (9.5%) | 205 (11.2%) | 59 (5.6%) |
| **Age groups**  16-29 | 156643 (32.3%) | 577 (34.0%) | 246 (23.4%) |
| 30-59 | 262958 (54.2%) | 876 (51.6%) | 615 (58.4%) |
| 60+ | 65474 (13.5%) | 244 (14.4%) | 192 (18.0%) |

^a^South east London Boroughs of Lambeth and Southwark; data provided by the UK Office for National Statistics

^b^Census sample are age 16 to 74 years and SELCoH sample are age 16 to 90; Frequencies may not add up to 100% due to missing values; percentages are unweighted

^c^Mixed ethnicity was not specified as a category in the SELCoH 1 study, but was included as a category in the SELCoH 2 study

Updated from Morgan et al (2014) doi:10.1192/bjp.bp.113.134452 with SELCoH 2 sample information

**Supplemental Table 2. Adjusted odds of CIS-R primary diagnoses by type of discrimination**

|  | **Depressive Episodes** | **Generalised Anxiety** | **Mixed Anxiety and Depression** |
| --- | --- | --- | --- |
|  | Adjusted OR, p-value | Adjusted OR, p-value | Adjusted OR, p-value |
| **Major discrimination** | |  |  |
| Any event | **1.9 (1.2-3.0), 0.01** | **1.1 (0.6-1.9), 0.69** | **1.1 (0.7-1.9), 0.66** |
| Fired | 1.5 (0.7-3.0), 0.26 | 1.5 (0.7-3.1), 0.27 | 0.6 (0.2-1.6),0.29 |
| Not hired | 1.1 (0.6-2.1), 0.78 | 1.0 (0.5-2.2), 0.96 | 2.1 (1.0-4.4), 0.04 |
| Denied promotion | 2.5 (1.3-4.9), 0.01 | 1.1 (0.4-2.8), 0.88 | 1.3 (0.6-3.0), 0.46 |
| In police treatment | 1.5 (0.8-2.8), 0.24 | 0.8 (0.3-1.9), 0.66 | 0.7 (0.3-1.8), 0.52 |
| In court system | 1.9 (0.8-4.6), 0.11 | 0.6 (0.1-2.4), 0.46 | 0.9 (0.3-2.5), 0.84 |
| In education | 1.5 (0.8-3.0), 0.25 | 1.2 (0.5-2.7), 0.72 | 1.1 (0.5-2.4), 0.73 |
| In housing | 0.3 (0.4-2.0), 0.20 | 0.6 (0.5-6.1), 0.65 | 3.2 (0.9-11.5), 0.07 |
| By neighbours | 2.8 (1.5-5.2), 0.001 | 0.7 (0.3-2.1), 0.59 | 0.9 (0.4-2.3), 0.86 |
| In bank services | 1.9 (0.7-4.9), 0.19 | 1.0 (0.2-4.6), 0.96 | 1.9 (0.8-4.3), 0.14 |
| In general services | 2.3 (1.2-4.8), 0.02 | 0.6 (0.2-1.9), 0.40 | 1.6 (0.6-4.1), 0.36 |
| In medical care | 2.1 (0.9-4.7), 0.06 | 0.9 (0.3-2.7), 0.87 | 1.1 (0.3-3.5), 0.89 |
| In public transport | 2.1 (1.1-4.1), 0.03 | 1.3 (0.5-3.0), 0.55 | 1.5 (0.7-3.5), 0.28 |
| **Anticipated discrimination** | |  |  |
| **Any event** | **2.1 (1.3-3.4), 0.002** | **1.9 (1.1-3.3), 0.03** | **0.7 (0.4-1.3), 0.27** |
| Avoid applying for education or work | 1.9 (1.1-3.4), 0.03 | 1.2 (0.6-2.5), 0.64 | 1.1 (0.6-2.2), 0.72 |
| Avoid health service contact | 1.7 (0.7-3.9), 0.24 | 1.4 (0.4-4.4), 0.60 | 1.7 (0.6-4.9), 0.35 |
| Avoid neighbourhoods | 1.7 (0.9-3.0), 0.08 | 1.4 (0.8-2.7), 0.26 | 0.9 (0.4-1.8), 0.79 |
| **Everyday discrimination** | |  |  |
| **Median score and above** | **2.8 (1.6-4.7), <0.001** | **1.3 (0.8-2.3), 0.30** | **0.9 (0.5-1.6),0.82** |

Model adjusts for age, gender, sexual identification, ethnicity, number of years in the UK, English as 1st language, relationship status,

education, employment status, ethnic identity and common mental disorder at SELCoH 1
